# Artesunate for the treatment of severe malaria: A retrospective review of patients admitted to two tertiary hospital intensive care units in Johannesburg, South Africa

**DOI:** 10.4102/sajid.v35i1.174

**Published:** 2020-12-18

**Authors:** Ratidzo Tadzimirwa, Shahed Omar, Jacqueline M. Brown, Ismail S. Kalla

**Affiliations:** 1 Department of Internal Medicine, Faculty of Health Sciences, School of Clinical Medicine, University of the Witwatersrand, Johannesburg, South Africa; 2 Critical Care (ICU), Faculty of Health Sciences, School of Clinical Medicine, Chris Hani Baragwanath Academic Hospital, University of the Witwatersrand, Johannesburg, South Africa; 3 Pulmonology and Critical Care (ICU), Faculty of Health Sciences, School of Clinical Medicine, Charlotte Maxeke Johannesburg Academic Hospital, University of the Witwatersrand, Johannesburg, South Africa

**Keywords:** severe malaria, malaria, critically ill, artesunate, intensive care

## Abstract

**Background:**

Globally, malaria is one of the six major causes of deaths from communicable diseases. In South Africa, malaria is known to be endemic in three provinces. Two large trials, AQUAMAT and SEAQUAMAT, demonstrated the superiority of intravenous (IV) artesunate compared to quinine. A systematic review (including the above trials) demonstrated a mortality benefit for adult patients treated with artesunate, but included studies that were conducted in Asia with no adult data available for Africa. Given the lack of local data, we conducted this study to investigate the use of artesunate for the treatment of severe malaria at two academic adult intensive care units (ICUs) in Johannesburg.

**Methods:**

We undertook a retrospective patient record review. All patients admitted to the two ICUs and treated for severe malaria using artesunate were included. The study period extended from April 2010 to April 2014. The primary outcome was to determine the observed mortality and relate it to the predicted mortality based on the Acute Physiology and Chronic Health Evaluation (APACHE II) severity of illness score. The ratio of the observed mortality to the expected mortality based on the APACHE II severity of illness score provides a standardised mortality ratio (SMR). Clinical and laboratory parameters data were analysed.

**Results:**

There were 56 patients included in the study, of which 40 were male (71.4%). The mean APACHE II score was 19 (standard deviation 5.4). We observed a lower than predicted mortality rate of 21.4% (SMR 0.66). Human immunodeficiency virus (HIV) was the most prevalent comorbidity (32%). There was no travel history in 26.8% of patients. Heart rate, respiratory rate and Glasgow Coma Scale (GCS) all improved significantly from admission to the time of discharge (*p* ≤ 0.01). Acidaemia, bilirubin, urea and bleeding risk (platelet count) also improved (*p* ≤ 0.01). Mechanical ventilation was associated with an increased risk of death (OR 35; CI 7.0–182).

**Conclusion:**

In this retrospective two-centre study, IV artesunate was associated with a lower than predicted mortality in adult patients with severe malaria requiring ICU admission.

## Background

According to the 2013 World Health Organization (WHO) World Malaria Report, malaria is a major cause of mortality and morbidity in malaria-endemic areas of the world. In 2018, 228 million cases of malaria were estimated to have been reported throughout the world with 85% of cases in sub-Saharan Africa and India.^1^ This makes malaria the second most common cause of infectious disease-related deaths in the world, after tuberculosis.^2^

Malaria is prevalent throughout most of the tropical regions of the world.^3^ *Plasmodium falciparum* predominates in Africa, New Guinea, the Dominican Republic and Haiti. *Plasmodium vivax* is more common in Central America and Asia.^4^

In South Africa, malaria is known to be endemic in three provinces, namely Mpumalanga, KwaZulu-Natal and Limpopo.^5^ The highest transmission occurs in north-eastern Limpopo whilst Mpumalanga has recently seen a significant reduction in cases.^6^ Over seven thousand (7626) cases of malaria were reported throughout South Africa in 2011.^7^ Globally, malaria is one of the six major causes of deaths from communicable diseases and locally there were between 10 000 and 30 000 notified cases of malaria per year in South Africa (2015–2019).^8^ Malaria cases and related deaths are also reported in Gauteng and attain a peak during the rainy summer season. This is because of travellers returning from malaria-endemic areas during the summer holidays.^7^

Although quinine has been the mainstay of treatment for severe malaria, complications such as quinine-induced hyperinsulinaemia, hypoglycaemia, arrhythmias, sterile abscesses and painful intramuscular (IM) injection, make it less desirable. Furthermore, the SEAQUAMAT trial showed mortality benefit with the use of intravenous (IV) artesunate over quinine and these findings were replicated by the AQUAMAT trial in African children (excluding South Africa).^9,10^ It is noteworthy that a systematic review that demonstrated a mortality benefit for adult patients treated with artesunate included studies that were conducted in Asia with no adult data from Africa.^11^ This may be important as the adult mortality (Asia) was higher than the paediatric mortality (mainly from Africa).

Artesunate was an unregistered drug for treating severe malaria in South Africa during the study period and was used under Section 21 of *the Medicines and Related Substances Act* and the Parenteral Artesunate Access Programme.^12^ It has subsequently been registered with the South African Health Products Regulatory Authority (SAHPRA) during 2017. Given the lack of local data regarding the treatment of severe malaria in critically-ill adult patients using artesunate, we decided to evaluate its efficacy.

## Methods

### Study design and setting

This was a retrospective, descriptive study conducted at two tertiary-level academic intensive care units (ICUs) in Johannesburg, which is a non-endemic area for malaria.

Population, sample and anti-malarial drug treatment:

Data belonging to all patients admitted to the ICUs of Chris Hani Baragwanath Academic Hospital and Charlotte Maxeke Johannesburg Academic Hospital with a diagnosis of severe malaria, treated with IV artesunate, between 01 April 2010 and 30 April 2014 were included. All these patients participated in the Parenteral Artesunate Access Programme.^12^ Parenteral artesunate had been recommended for use in preference to quinine for the treatment of severe malaria, given its significant mortality and safety benefits. As the product had not yet been registered for use in South Africa, the Parenteral Artesunate Access Programme was launched to reduce malaria-related mortality. Pregnant patients were also treated in the maternity high-care unit and this meant that our data were likely to be incomplete for the period under investigation for this group. Artesunate was used intravenously initially and oral doxycycline was added as soon as the patient’s enteral tolerance was established. IV artesunate was used for a minimum of 48 h and a maximum of 7 days followed by oral artemether or lumefantrine for 3 days.

### Outcomes

The primary outcome was to determine the standardised mortality ratio (SMR). This is the observed mortality, related to the predicted mortality based on the Acute Physiology and Chronic Health Evaluation (APACHE) II severity of illness score. An SMR less than 1 indicates a better than predicted outcome, whilst an SMR greater than 1 indicates mortality worse than expected.^13^ Secondary outcomes included the clinical changes from admission to discharge, the changes in the indicators of severe malaria between admission and discharge, differences between survivors and non-survivors and the frequency of WHO indicators of severe malaria.

### Data collection

Collection of data from patients’ ICU records was carried out using a standardised data collection sheet. These data were then entered into an electronic spreadsheet. No identifying data were captured.

### Statistical analysis

We used Statistica v13 for analysis.^14^ All data were assessed for normality. Normally-distributed data were described using mean and standard deviation (SD), whilst non-normally-distributed data used medians and interquartile ranges (IQR, Q3–Q1). Continuous variables were compared using Wilcoxon’s matched pairs test or *T*-test for dependent variables depending on the distribution. Proportions and percentages were compared using the Chi square test and confidence intervals for the SMR were calculated using the Mid P test.

## Results

There were 56 patients admitted with severe malaria to two academic ICUs during the 49-month study period. Table 1 provides a baseline description of the patients.

**TABLE 1:** Patients description.

Variable	%	Mean	SD	Median	IQR	*n*
Age	-	40.3	11.6	-	-	56
Gender (male)	71.4	-	-	-	-	40/56
Weight (kg)	-	73.0	15.3	-	-	44
Parasitaemia % (*P. falciparum*)	-	-	-	10.6	13.2	44
APACHE II	-	19.0	5.4	-	-	35
Predicted mortality	32.2	-	-	-	-	-

SD, standard deviation; IQR, Interquartile range; APACHE II, Acute Physiology and Chronic Health Evaluation.

Nearly half of the patients had at least one co-morbidity (48.2%). The most common co-morbidity was human immunodeficiency virus (HIV) (32.1%) (see Table 2).

**TABLE 2:** Past medical history.

Number	Description	*N*	%
1	No known co-morbidities	29	51.8
2	HIV positive	18	32.1
3	Any other co-morbidities	9	16.1

HIV, human immunodeficiency virus.

**FIGURE 1:**
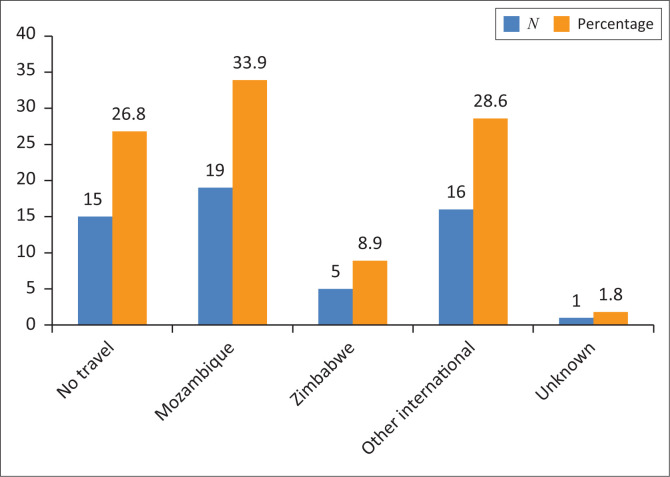
Travel history.

Travel outside of Johannesburg, to a malaria-endemic region within the past 30 days was recorded. Figure 1 gives the regions and distribution of patients travelling to these regions. Of note, 26.8% of patients had no history of travel to a malaria-endemic region. The most commonly visited malaria-endemic region was Mozambique (33.9%) (see Figure 1).

### Primary outcome

The mean APACHE II score was 19 (5.4 SD). The predicted mortality for this group of patients was 32.2%. We observed a lower than predicted mortality rate of 21.4% (12/56). The SMR was 0.66 (CI 0.45–0.96). There was no significant difference in the mortality rates of those with a travel history (9/40, 22.5%) versus those with no travel history (2/15, 13%), *p* = 0.365 (Fisher’s exact test).

### Secondary outcomes

Heart rate, respiratory rate and Glasgow Coma Scale (GCS) all improved from admission to discharge. These improvements were clinically and statistically significant (See Table 3). Systolic blood pressure increased from 105 mm Hg at admission to 118 mm Hg at discharge, but this was not statistically significant.

**TABLE 3:** Clinical characteristics compared from admission (D1) to discharge or death (DC) using Wilcoxon matched pairs test or *T*-test for dependent samples.

Parameter	D1				Discharge				*N*	*p*
	Mean	Median	SD	IQR	Mean	Median	SD	IQR		
Heart rate/min	108	-	21.4	-	98	-	24.5	-	55	0.001
Respiratory rate	27	-	9.4	-	23	-	7.2	-	53	0.007
GCS below 15	-	3	-	6	-	0	-	2	38	0.000
Systolic blood pressure	-	105	-	50	-	118	-	45	54	0.550

SD, standard deviation; IQR, Interquartile range; GCS, Glasgow Coma Scale.

There were statistically significant improvements in several indicators of severe malaria between admission and discharge. Acidaemia, total bilirubin, markers of acute kidney injury (AKI) and bleeding risk (platelet count) all improved (see Table 4).

**TABLE 4:** Changes in the indicators of severe malaria from admission (D1) to discharge or death (DC) using Wilcoxon matched pairs test or *T*-test for dependent samples.

Parameter	D1		Discharge		*N*	*p*
	Median	IQR	Median	IQR		
TCO_2_† meq/L	17	8	23	7	50	0.000
Total bilirubin µmol/L	63	101	53	88	34	0.013
Urea mmol/L	22.4	17.2	11.5	10.4	52	0.000
Platelets 10^3^/µL	48	56	89.5	92	53	0.000

† , TCO_2_- total carbon dioxide (metabolic state).

IQR, Interquartile range.

### Survivors versus non-survivors

Non-survivors had a lower GCS and more severe metabolic acidosis on admission (D1). There was a trend towards a lower parasitaemia in the non-survivors. As expected, non-survivors had a higher APACHE II score (see Table 5). Patients who were mechanically ventilated had an increased risk of death (odds ratio (OR) 35, CI 7.0–182). Similarly, patients requiring vasopressors were also at increased risk of death (OR 4.3, CI 1.7–11.4).

**TABLE 5:** Differences between survivors and non-survivors using the Mann–Whitney *U* test or *T*-test for independent samples.

Parameter	Non-survivors				Survivors				*N*	*p*
	Median	IQR	Mean	SD	Median	IQR	Mean	SD		
GCS below total score	7.5	2.5	-	-	2.0	5	-	-	44	0.000
†TCO_2_ meq/L	13	8.5	-	-	18.5	6	-	-	40	0.010
Parasitaemia %	6.6	10.0	-	-	11.2	14.3	-	-	33	0.053
APACHE II	-	-	22.5	4.9	-	-	18.2	5.3	35	-

† , TCO_2_ – total carbon dioxide.

SD, standard deviation; IQR, Interquartile range; GCS, Glasgow Coma Scale; APACHE II Acute Physiology and Chronic Health Evaluation.

Factors associated with disease severity and prognosis include seizures and hypoglycaemia, and are provided in Table 6.^14^ There were seven patients with seizures during their ICU stay. Four patients had single seizures, two patients had two seizures and one had four seizures. No patient met the strict definition of hypoglycaemia (glucose level < 2.2 mmol/L) as standard management institutes corrective action at levels of 3.5 mmol/L.

**TABLE 6:** Documented World Health Organization indicators^15^ of severe malaria and poor prognosis.

Number	Complication	*N*	Frequency (%)
1	Anaemia (Hb ≤ 7.0 g/dL)	14	28.6
2	AKI requiring RRT	28	49
3	Mechanical ventilation (ARDS)	21	30
4	Hypoglycaemia (HGT < 3.5 mmol/L)	3	5.5
5	Shock (vasopressor use)	16	31
6	†Possible DIC (platelets ≤ 25 × 10^3^/µL)	7	12.5
7	Seizures	7	12.5
8	pH ≤ 7.25	21	37.5

† , We used platelet count as an indicator for DIC because of the lack of objective data regarding bleeding risk.

AKI, acute kidney injury, ARDS, Acute respiratory distress syndrome; DIC, Disseminated intravascular coagulation; HGT, Hemo Glucose Test; RRT, Renal replacement therapy.

## Discussion

The main finding of our study was a lower than expected mortality of 21.4% (expected 32.2%, SMR 0.66). This compares well with the large SEAQUAMAT trial, the largest adult randomised clinical trial comparing IV artesunate and quinine for severe falciparum malaria. It included 1461 patients, of which 86% (1259) were adults. The mean age in this study was 27.9 years. The acidaemia and renal dysfunction were less severe than our study data, median TCO_2_ of 22 mmol/L and median area of 9.2 to 10.4 mmol/L. The utilisation of organ support was also low: renal replacement therapy 8%, vasopressor support 3% and mechanical ventilation 4% – 5%. The overall mortality for the study was 19%.^7^

Our group comprised an older cohort (mean age 40.3 years) with almost half having at least one co-morbidity (48.2%) and 32.2% being HIV positive. Our group had more significant acidaemia (median TCO_2_ was 17 mmol/L at the time of admission) and a greater level of renal dysfunction (median urea 22.4 mmol/L at the time of admission). In addition, our group required significantly more organ support: renal replacement therapy 49%, vasopressor support 31% and mechanical ventilation 30%. Despite these differences, the mortality in our group was similar (21.4%). It appears that in critically ill patients with severe falciparum malaria, the benefit of IV artesunate may be greater than anticipated.

Odyssean malaria is defined as malaria in a person with no history of recent travel to a malaria-endemic region. It is also colloquially known as airport, suitcase, minibus or taxi-rank malaria. It is distinct from imported malaria^16^. It is plausible and likely that an *Anopheles* malaria vector mosquito has been inadvertently transported from a malaria-endemic area to a non-malaria-endemic region and infects local residents.^16^ A study in the Gauteng Province of South Africa described 46 cases of odyssean malaria over an 8-year period between 1996 and 2004. The case fatality rate of this group was 13%.^17^ This was about 10 fold higher than the national case fatality rate of between 0.6% and 1.0%.^16^ This may in part be explained by the delay in diagnosis and the non-immune status of these patients.^15,16,17^

Our data confirm the significance of odyssean malaria in South Africa and particularly in Gauteng where our study was conducted. We found that 26.8% of our malaria cases had no documented travel history. Although we did not perform in-depth family interviews, the numbers we found over the 4 years of the study are in keeping with that reported by Frean et al.^17^ However, our group with no documented travel history is different from the odyssean malaria group in the above-mentioned study. Our cohort comprises critically ill patients with multi-organ dysfunction and an expected mortality of approximately 30%. Although we found no significant difference in mortality between those without a travel history (13%) compared to those with a travel history (22%), there appears to be a trend. Note that the mortality of 13% in our study is not dissimilar to 13% in the study by Frean et al. What is clearly different is the comparator mortality in the two studies. Our study compares 13% mortality to 22% for the group with travel history. It is important to note that our group comprises only cases of severe malaria and the overall mortality is naturally higher. The study by Frean et al. uses the national case fatality rate for comparison. This is obviously much lower, around 1% as it includes all malaria cases, the majority of which are not severe malaria. Additionally, the group of Frean et al. does not provide for severity of illness. Our data are limited by the retrospective nature of the study and factors such as undocumented (‘illegal’) migrants who may have withheld their travel history, or a thorough travel history not being taken from critically ill patients cannot be excluded. Despite the limitations of our study, our findings do challenge the current thinking regarding the impact of odyssean malaria on mortality, and this needs further research.

Regarding the possible WHO indicators of severe malaria and possible adverse effects, although we found three episodes of glucose levels of ≤ 3.5 mmol/L in our study, there were no episodes of hypoglycaemia (≤ 2.2 mmol/L). This is in contrast to 26% hypoglycaemia (≤ 2.2 mmol/L) described by Mehta et al.^18^ The absence of hypoglycaemia in our study reflects the additional safety component of using artesunate as compared to quinine. This comparison is reasonable as the incidences of organ dysfunction between the cohort of Mehta et al.^18^ and our study cohort were similar: AKI (49% vs. 49%), shock (28% vs. 31%), acute respiratory distress (36% vs. 30%) and disseminated intravascular coagulation (11% vs. 12.5%), respectively. A systematic review also confirmed the advantage of artesunate over quinine with regards to the occurrence of hypoglycaemia.^11^ The study by Mehta et al. also found a significant number of instances of inappropriate administration of quinine reflecting more complicated dosing for this drug.^18^

A negative effect observed by other investigators when comparing artesunate to quinine, was the higher risk of neurological sequelae with the use of artesunate.^11^ We found a seizure rate of 12.5% in our study, where only artesunate was used. The study by Mehta et al. where quinine was used found a seizure rate of 24%.^18^ Our data therefore suggest a lower incidence of seizures when compared to quinine. All other organ dysfunction was similar in the two studies as previously described.

When comparing travel history with other data from South Africa, we found that the most frequent travel was to Mozambique. This was indeed the most commonly visited country in a study of over 2000 malaria patients as stated by Perovic et al.^19^ Almost 34% of our group had a history of travel to Mozambique.

Finally with regards to HIV infection, it was the most common co-morbidity in our study (32%): twice that of all other co-morbidities combined. Previous data between 2002 and 2004 from South Africa observed HIV in 53% of cases.^18^ This study also revealed that patients with HIV were more likely to have a delayed presentation possibly due to seeking care from a traditional health care worker prior to presentation. When comparing survivors to non-survivors, severity of illness (APACHE II), parasitaemia, acidosis, GCS, ARDS and vasopressor use (shock) were the differentiators in our study, but not HIV status.

### Limitations

This was a retrospective study with a small number of patients from only two centres. In addition, the use of SMR, as opposed to a control group using quinine and the availability of only 36/56 outcomes, are limitations to the study. Despite these limitations, it is the only study we are aware of that evaluated the efficacy of artesunate in critically ill patients admitted to ICU in South Africa.

## Conclusion

In this retrospective two-centered study, IV artesunate was associated with a lower than predicted mortality in adult patients with severe malaria requiring ICU admission.

## References

[1] World Health Organization. World Malaria Report 2019 [homepage on the Internet]. Geneva: World Health Organization; 2019 [cited 2020 Aug 09]. Available from: https://www.who.int/publications-detail-redirect/9789241565721

[2] Schantz-Dunn J, Nour NN. Malaria and pregnancy: A global health perspective. Rev Obstet Gynecol. 2009;2(3):186. PMC2760896 19826576

[3] Longo D, Fauci A, Kasper D, Hauser S, Jameson J, Loscalzo J. Harrison’s principals of internal medicine. 18th ed. New York, NY: Mcgraw-Hill Professional; 2011.

[4] Gething PW, Elyazar IR, Moyes CL, et al. A long neglected world malaria map: *Plasmodium vivax* endemicity in 2010. PLoS Negl Trop Dis. 2012;6(9):e1814. 10.1371/journal.pntd.0001814 22970336 PMC3435256

[5] National Guidelines for the prevention of malaria, South Africa, 2018 [homepage on the Internet]. NICD [cited 2020 Jul 21]. Available from: https://www.nicd.ac.za/wp-content/uploads/2019/03/National-Guidelines-for-prevention-of-malaria_updated-08012019-1.pdf

[6] Malaria Update: NICD September 2019 [homepage on the Internet]. [cited 2020 Jul 21]. Available from: https://www.nicd.ac.za/malaria-update-september-2019/

[7] Moonasar D, Asomugha C, Baker L, et al. Malaria risk – Be warned! S Afr Med J. 2011;101(12):865–867. 22273020

[8] Malaria [homepage on the Internet]. NICD; 2019. [cited 2020 Jul 21]. Available from: https://www.nicd.ac.za/diseases-a-z-index/malaria/

[9] White N, Dondorp AM, South East Asian Quinine Artesunate Malaria Trial (SEAQUAMAT) Group. Artesunate versus quinine for treatment of severe falciparum malaria: A randomised trial. Lancet. 2005;366(9487):717–725. 10.1016/S0140-6736(05)67176-0 16125588

[10] Dondorp AM, Fanello CI, Hendriksen IC, et al. Artesunate vs quinine in the treatment of severe falciparum malaria in African children (AQUAMAT): An open-label, randomised trial. Lancet. 2011;376(9753):1647–1657. 10.1016/S0140-6736(10)61924-1 PMC3033534 21062666

[11] Sinclair D, Donegan S, Isba R, Lalloo DG. Artesunate versus quinine for treating severe malaria. Cochrane Database Syst Rev. 2012;(6):CD005967. 10.1002/14651858.CD005967.pub4 22696354 PMC6532684

[12] Kift EV, Kredo T, Barnes KI. Parenteral artesunate access programme aims at reducing malaria fatality rates in South Africa. S Afr Med J. 2011;101(4):240–241. 10.7196/SAMJ.4761 21786724

[13] Kute VB, Shah PR, Munjappa BC, et al. Outcome and prognostic factors of malaria-associated acute kidney injury requiring hemodialysis: A single center experience. Indian J Nephrol. 2012;22(1):33–38. 10.4103/0971-4065.83737 22279340 PMC3263060

[14] TIBCO Software Inc. Statistica (data analysis software system), version 13. http://statistica.io [computer program]. Palo Alto: TIBCO Software Inc; 2017.

[15] Trampuz A, Jereb M, Muzlovic I, Prabhu RM. Clinical review: Severe malaria. Crit Care. 2003;7(4):315–323. 10.1186/cc2183 12930555 PMC270697

[16] Dlamini SK. Diagnosis and treatment of imported and odyssean malaria. S Afr Med J. 2014;104(5):344. 10.7196/SAMJ.8306 25212209

[17] Frean J, Brooke B, Thomas J, Blumberg L. Odyssean malaria outbreaks in Gauteng Province, South Africa, 2007–2013. S Afr Med J. 2014;104(5):335–338. 25212198 10.7196/samj.7684

[18] Mehta U, Durrheim DN, Blumberg L, et al. malaria deaths as sentinel events to monitor healthcare delivery and antimalarial drug safety. Trop Med Int Health. 2007;12(5):617–628. 10.1111/j.1365-3156.2007.01823.x 17445129

[19] Perovic O, Crewe-Brown HH, Blumberg L, et al. Malaria at the Chris Hani Baragwanath Hospital, Soweto. S Afr Med J. 2000;90(4):365–366. 10957920

